# Molecular and microscopic analysis of the gut contents of abundant rove beetle species (Coleoptera, Staphylinidae) in the boreal balsam fir forest of Quebec, Canada

**DOI:** 10.3897/zookeys.353.5991

**Published:** 2013-11-20

**Authors:** Jan Klimaszewski, Marie-Josee Morency, Philippe Labrie, Armand Séguin, David Langor, Timothy Work, Caroline Bourdon, Evelyne Thiffault, David Paré, Alfred F. Newton, Margaret K. Thayer

**Affiliations:** 1 Natural Resources Canada, Canadian Forest Service, Laurentian Forestry Centre, 1055 du P.E.P.S., P.O. Box 10380, Stn. Sainte-Foy, Québec, Québec, G1V 4C7, Canada; 2 Natural Resources Canada, Canadian Forest Service, Northern Forestry Centre, 5320-122 Street, Edmonton, Alberta, T6H 3S5, Canada; 3 Département des sciences biologiques, Université du Québec à Montréal, CP 8888, Succursale Centre-ville, Montréal, Québec, H3C 3P8, Canada; 4 Integrative Research Center, The Field Museum of Natural History, Chicago, Illinois, 60605-2496, U.S.A.

**Keywords:** Rove beetles, Staphylinidae, Coleoptera, diet, fungivory, mycophagy, gut analysis, trophic relationship, saproxylic, boreal forest, Canada, Ascomycota, Basidiomycota, bacteria

## Abstract

Experimental research on beetle responses to removal of logging residues following clearcut harvesting in the boreal balsam fir forest of Quebec revealed several abundant rove beetle (Staphylinidae) species potentially important for long-term monitoring. To understand the trophic affiliations of these species in forest ecosystems, it was necessary to analyze their gut contents. We used microscopic and molecular (DNA) methods to identify the gut contents of the following rove beetles: *Atheta capsularis* Klimaszewski, *Atheta klagesi* Bernhauer, *Oxypoda grandipennis* (Casey), *Bryophacis smetanai* Campbell, *Ischnosoma longicorne* (Mäklin), *Mycetoporus montanus* Luze, *Tachinus frigidus* Erichson, *Tachinus fumipennis* (Say), *Tachinus quebecensis* Robert, and *Pseudopsis subulata* Herman. We found no apparent arthropod fragments within the guts; however, a number of fungi were identified by DNA sequences, including filamentous fungi and budding yeasts [Ascomycota: *Candida derodonti* Suh & Blackwell (accession number FJ623605), *Candida mesenterica* (Geiger) Diddens & Lodder (accession number FM178362), *Candida railenensis* Ramirez and Gonzáles (accession number JX455763), *Candida sophie-reginae* Ramirez & González (accession number HQ652073), *Candida* sp. (accession number AY498864), *Pichia delftensis* Beech (accession number AY923246), *Pichia membranifaciens* Hansen (accession number JQ26345), *Pichia misumaiensis* Y. Sasaki and Tak. Yoshida ex Kurtzman 2000 (accession number U73581), *Pichia* sp. (accession number AM261630), *Cladosporium* sp. (accession number KF367501), *Acremoniumpsammosporum* W. Gams (accession number GU566287), *Alternaria* sp. (accession number GU584946), *Aspergillus versicolor* Bubak (accession number AJ937750), and *Aspergillusamstelodami* (L. Mangin) Thom and Church (accession number HQ728257)]. In addition, two species of bacteria [*Bradyrhizobium japonicum* (Kirchner) Jordan (accession number BA000040) and *Serratia marcescens* Bizio accession number CP003942] were found in the guts. These results not only provide evidence of the consumer-resource relations of these beetles but also clarify the relationship between rove beetles, woody debris and fungi. Predominance of yeast-feeding by abundant rove beetles suggests that it may play an important role in their dietary requirements.

## Introduction

Rove beetles (Coleoptera: Staphylinidae) have proven to be useful indicators of forest disturbance and recovery because they are sensitive to environmental perturbations, diverse in species and trophic roles, easily sampled, and at least in central Europe and Canada, mostly readily identified using a wealth of available taxonomic tools (Boháč 1990, 1999, Pohl et al. 2008). Many staphylinid species show distinct response patterns following forest disturbances (e.g., Pohl et al. 2007, 2008, Klimaszewski et al. 2008, Work et al. 2013). For example, in a recent study of rove beetles following removal of logging residues by whole-tree harvesting in boreal balsam fir forests of Quebec, three *Atheta* species, *Tachinus fumipennis* (Say) and *Tachinus frigidus* Erichson were negatively affected by the removal of forest biomass, while *Gabrius brevipennis* (Horn), *Pseudopsis subulata* Herman and *Quedius labradorensis* Smetana were not and their catch increased (Work et al. 2013). While studies comparing species assemblages can quantify the overall effects of harvest treatments or other forest disturbances, they are often not designed to identify specific underlying mechanisms for individual species’ responses. Study of trophic roles may provide useful insights into these response patterns by assessing factors such as individual predator-prey (or consumer-resource) relationships, the degree of diet specialization, and possible associations of beetles with specific microhabitats that may serve as habitat or substrate for their food resources.

Rove beetles are a diverse group exhibiting a wide variety of trophic relationships and occupying numerous microhabitats in forest ecosystems. Many Aleocharinae and Staphylininae, e.g., species of *Aleochara*, *Philonthus*, *Platydracus*, and *Staphylinus*, are voraciouspredators of other arthropods such as fly larvae (Klimaszewski 1984, Smetana 1995). At least some species of Scaphidiinae, Osoriinae, Tachyporinae, and Aleocharinae (Gyrophaenina) eat the flesh or spores of fungal sporocarps (Seevers 1951, Ashe 1984, Newton 1984). Oxytelinae are generally detritivores and feed on decaying plant material and algae (Thayer 2005, Makranczy 2006). A few Omaliinae, e.g. *Eusphalerum* and some other genera, are pollen-feeders (Thayer 1987, 2005). A few species, such as the aleocharine *Himalusa thailandensis* Pace, Klim. & Cent., feed on live plant tissue (Klimaszewski et al. 2010). Most information on food sources of rove beetles has been obtained through observation of individual beetles in the field or laboratory, or inference from habitat associations of species. For example, some groups (e.g., *Aleochara*, *Philonthus*) that are collected in decaying mushrooms are predators of dipteran larvae that co-occur within these fungi (Klimaszewski 1984, Smetana 1995). While direct observations of feeding provide compelling evidence of dietary preferences, inferences based on habitat preferences are not definitive evidence of consumer-resource relationships. Other methods have been used to more definitively establish feeding habits of beetles including microscopical examination of gut contents (Newton 1984, Thayer 1987) and immunological methods (Dennison and Hodkinson 1983). The use of molecular techniques to investigate dietary preferences and trophic links in rove beetles is presented here for the first time, but similar techniques were used in the past to investigate invertebrate predators for multiple prey using DNA targets (Harper et al. 2005). Increasingly large databases of DNA sequences in repositories such as GenBank and MycoBank will make these techniques more and more useful for examining relationships between beetles and cryptic food items such as fungi and bacteria (Crous et al. 2004, Robert et al. 2005). For beetles that feed on organisms with relatively strict habitat requirements, such as fungal species that require lignocellulose, molecular gut analyses may lead to inferences on the importance of habitat elements such as downed deadwood (Suh and Blackwell 2005a, b).

In this study we use both microscopic examination and molecular analysis of gut contents to more precisely characterize the feeding habits and trophic role of 10 rove beetle species common in the boreal forest of Quebec. There are few published data on gut contents, of these species and little is known of their food affiliations, except for some general statements on habitat preferences of *Tachinus* species (Campbell 1973) and limited observations on hosts and gut contents of some *Tachinus* and *Pseudopsis* species (Newton 1984).

## Material and methods

### Sampling sites and rove beetle species

Rove beetles were collected as part of a large field experiment examining the impacts of biomass harvesting on forest ecosystem functioning (Thiffault et al. 2011, Venier et al. 2012) within the Montmorency Teaching and Research Forest (ranges of latitude and longitude: 47°13' to 47°22'N, and 71°05' to 71°11'W) approximately 70 km north of Quebec City, Quebec, Canada. This site is part of a 60-year-old boreal balsam fir-white birch dominated forest in the Laurentian Mountains. The site and experimental layout were described in detail by Work et al. (2013). All beetles were collected using pitfall traps deployed between June and August 2012. Beetles were collected from both harvested and unharvested stands in 75% ethanol with some vinegar, and later cleaned with 75 % ethanol and mounted on cards (Aleocharinae) or points (e.g., Tachyporinae, Pseudopsinae). We used the 10 most abundant rove beetle species for this study, together constituting 78% of all rove beetles collected (85–1785 specimens per species): Aleocharinae: *Atheta capsularis* Klimaszewski, *Atheta klagesi* Bernhauer, *Oxypoda grandipennis* (Casey); Tachyporinae: *Bryophacis smetanai* Campbell, *Ischnosoma longicorne* (Mäklin), *Mycetoporus montanus* Luze, *Tachinus fumipennis* (Say), *Tachinus frigidus* Erichson, *Tachinus quebecensis* Robert; and Pseudopsinae: *Pseudopsis subulata* Herman (Figs 2–11).

### Gut extraction for microscopical analysis

Six dried and mounted specimens of each species were selected from samples collected in 2012. Individual specimens were softened in distilled water and ammonia solution for about 15 minutes and their guts were dissected in distilled water under a stereoscopic microscope. The colon and rectum of the hindgut were transferred directly to absolute alcohol, placed on a glass slide with Canada balsam, and pressed by dissecting needles to liberate gut contents and then covered with a cover slip. Slides were studied under a compound microscope (Reichert, Vienna, Austria) and photographs were taken using an Olympus DP73 digital camera. The following publications were consulted for fungal spore illustrations: Hanlin (1990, 1998).

### Gut DNA extraction

DNA from gut contents was extracted from 10 individuals of each species of rove beetle using the QIAamp DNA Micro kit from Qiagen, according to the manufacturer’s specifications. Gut contents from the 10 individuals were pooled for DNA extraction. DNA samples were eluted from the columns in 100 µL of PCR grade nuclease-free water and the concentration was determined spectrophotometrically by reading absorbance at 260 nm and 280 nm with the Synergy Mx microplate reader (BioTek).

### PCR amplifications, cloning and sequence analysis

**Figure 1.**
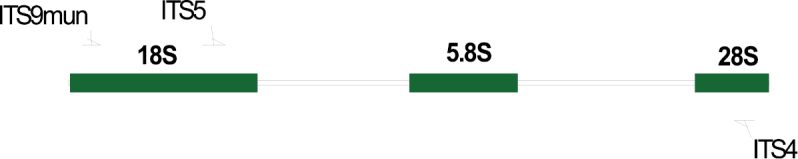
Map of ribosomal RNA genes and ITS regions.

PCR amplifications were performed using three primers universal to the internal transcribed spacer (ITS) regions of the nuclear ribosomal repeat and used in the following combinations (ITS9mun+ITS4 or ITS5+ITS4). The detailed sequences of the primers are given in Table 1; they specifically amplify a DNA fragment covering the ITS1 region, the 5.8S rRNA gene, and the ITS2 region between the 18S and 28S rRNA genes (Fig. 1).

**Table 1.** Primers used in this study.

Primer name	Primer sequence, 5’-3’	Primer source study
ITS9mun	TGTACACACCGCCCGTCG	Egger (1995)
ITS5	GGAAGTAAAAGTCGTAACAAGG	White et al. (1990)
ITS4	TCCTCCGCTTATTGATATGC	White et al. (1990)

**Figures 2–7.**
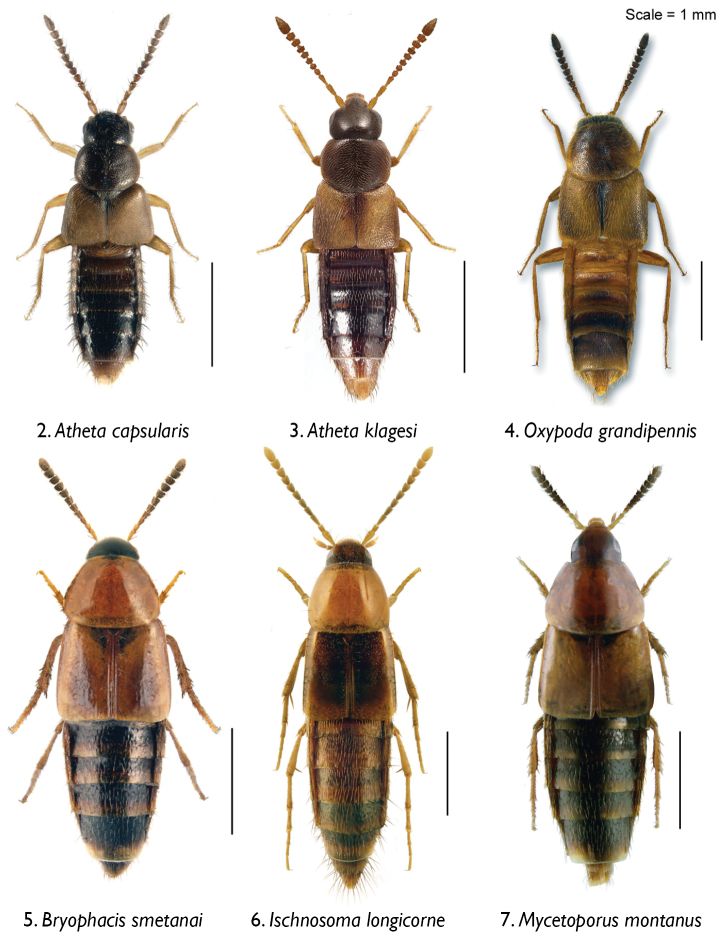
Body images of rove beetles in dorsal view: **2** *Atheta capsularis* Klimaszewski **3** *Atheta klagesi* Bernhauer **4** *Oxypoda grandipennis* (Casey) **5** *Bryophacis smetanai* Campbell **6** *Ischnosoma longicorne* (Mäklin) [previously cited as synonymous *Ischnosoma fimbriatum* Campbell] **7** *Mycetoporus montanus* Luze [previously cited as synonymous *Mycetoporus rugosus* Hatch].

**Figures 8–11.**
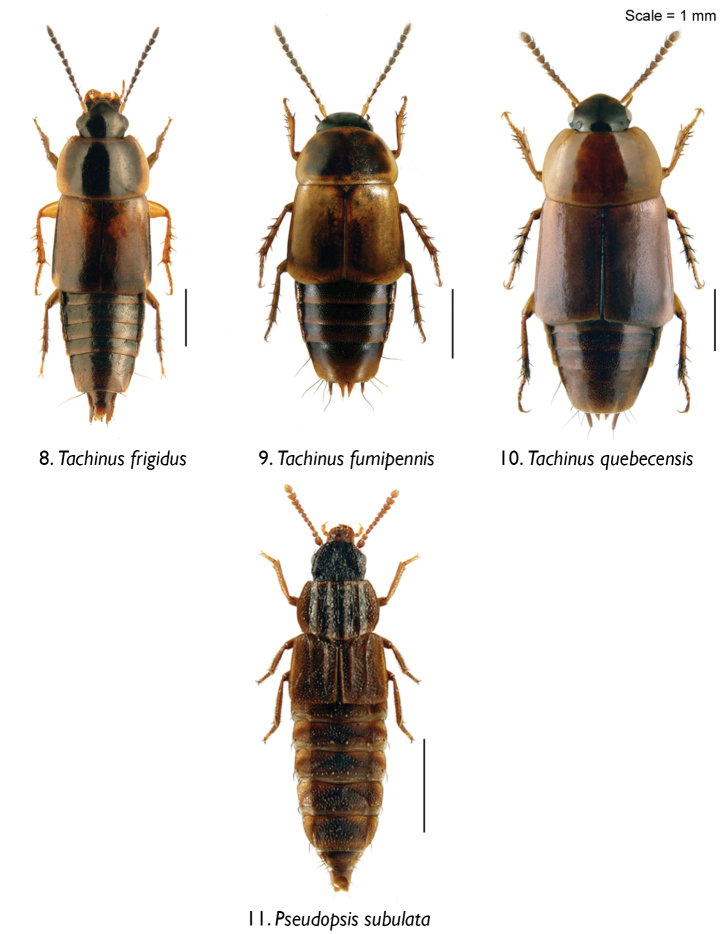
Body images of rove beetles in dorsal view: **8** *Tachinus frigidus* Erichson **9** *Tachinus fumipennis* (Say) **10** *Tachinus quebecensis* Robert **11** *Pseudopsis subulata* Herman.

The PCR reactions contained 30 ng of DNA, 2X HotStarTaq Plus Master Mix from Qiagen, which contains one unit of HotStarTaq Plus DNA Polymerase, PCR Buffer with 1.5 mM MgCl_2_, 200 μM of each dNTP and 0.3 μM of each primer in a 30 µL final reaction. PCR amplification was carried out using an initial denaturation step at 95°C for 15 min, followed by 35 cycles: 15s at 95°C, 30s at 52°C, 30s at 72°C, and a final extension for 10 min at 72°C. Cycling was performed on a PTC200 Peltier Thermal Cycler (MJ Research). Amplified fragments were inserted directly in the TA cloning vector (Invitrogen) and transformed into *Escherichia coli* strain DH10B. Plasmids were isolated using the Qiacube with the Qiagen miniprep columns (Qiagen) and sequenced with an ABI 3730xl Data Analyzer (Applied Biosystems). After removing the DNA cloning vector segments, the remaining sequences were compared with reference sequences contained in the GenBank nucleotide sequence database using the BLAST algorithm (Altschul et al. 1990) and in the MycoBank database search engine (Robert et al. 2005) to find the closest matching sequences. A total of 228 clones were sequenced in this study.

## Results

### Microscopic observations

**Figures 12–15.**
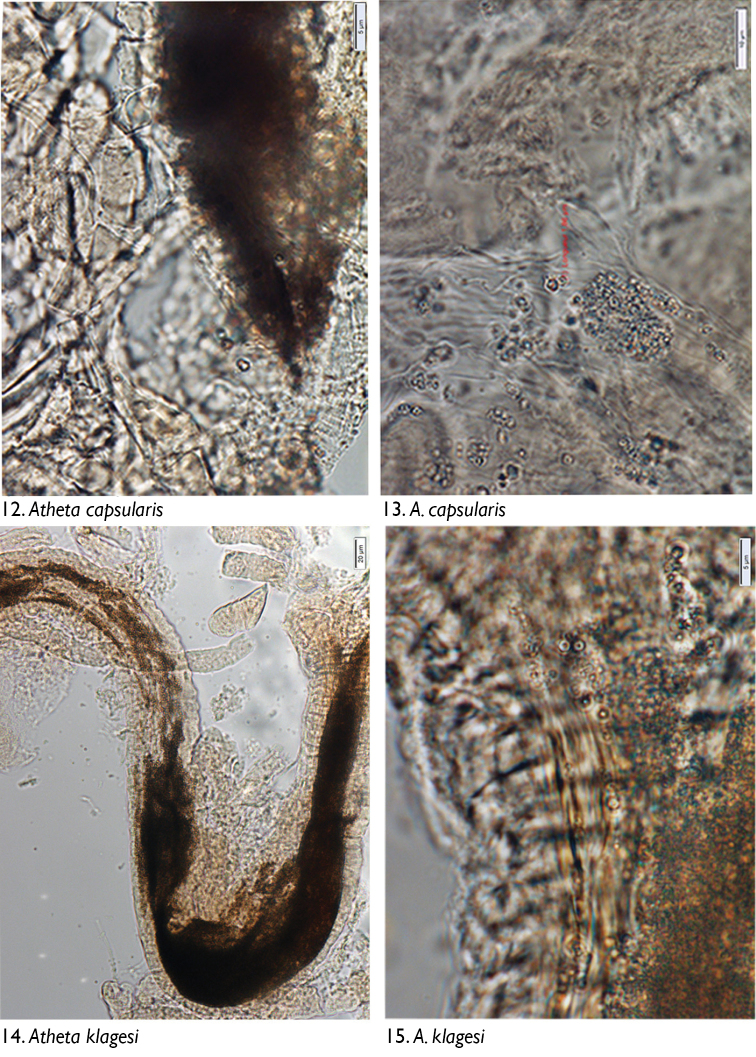
Images of hindgut content of the following rove beetle species: **12–13** *Atheta capsularis* Klimaszewski **14–15** *Atheta klagesi* Bernhauer.

**Figures 16–19.**
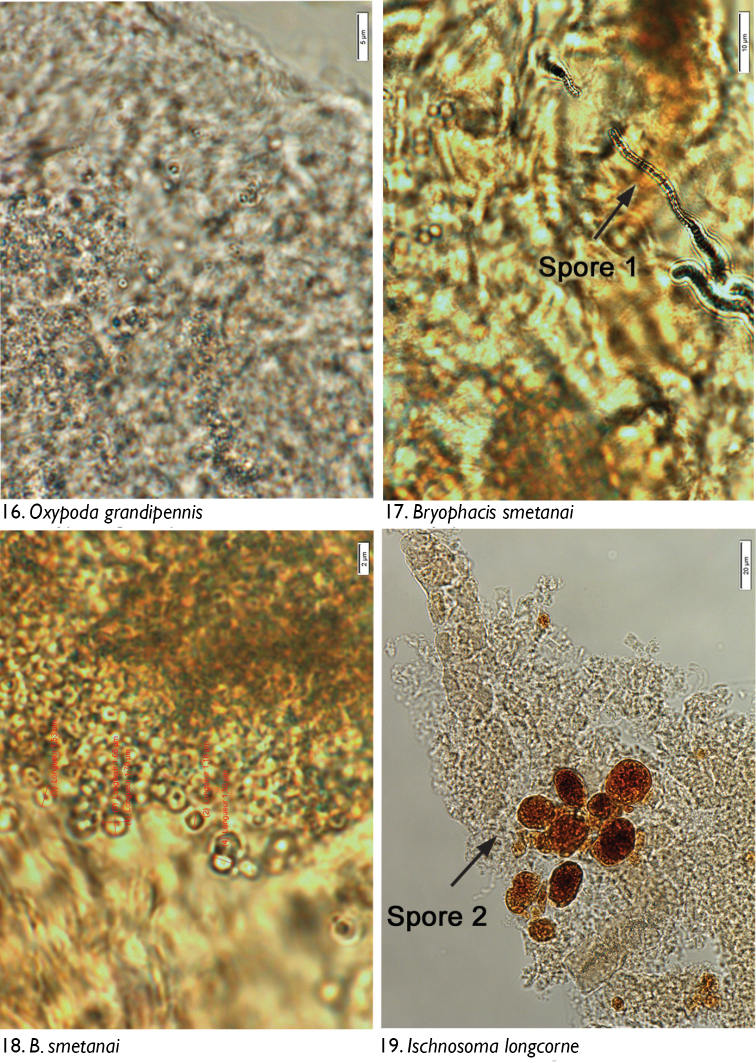
Images of hindgut content of the following rove beetle species: **16** *Oxypoda grandipennis* (Casey) **17–18** *Bryophacis smetanai* Campbell **19** *Ischnosoma longicorne* (Mäklin).

**Figures 20–23.**
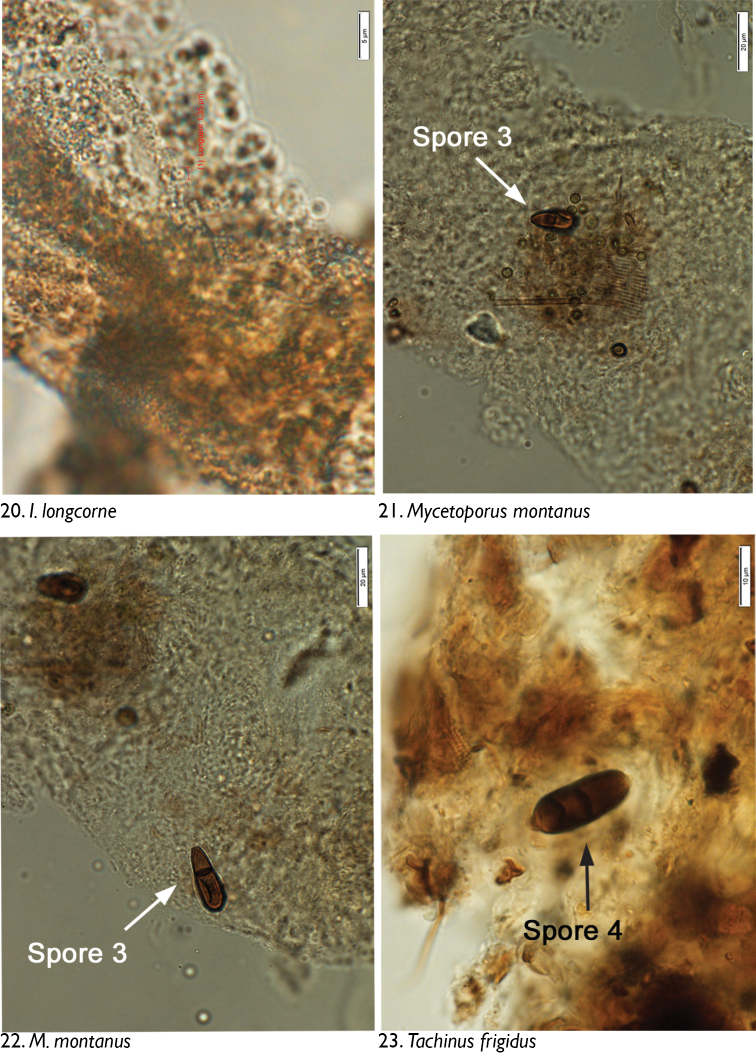
Images of hindgut content of the following rove beetle species: **20** *Ischnosoma longicorne* (Mäklin) **21–22** *Mycetoporus montanus* Luze **23** *Tachinus frigidus* Erichson.

**Figures 24–27.**
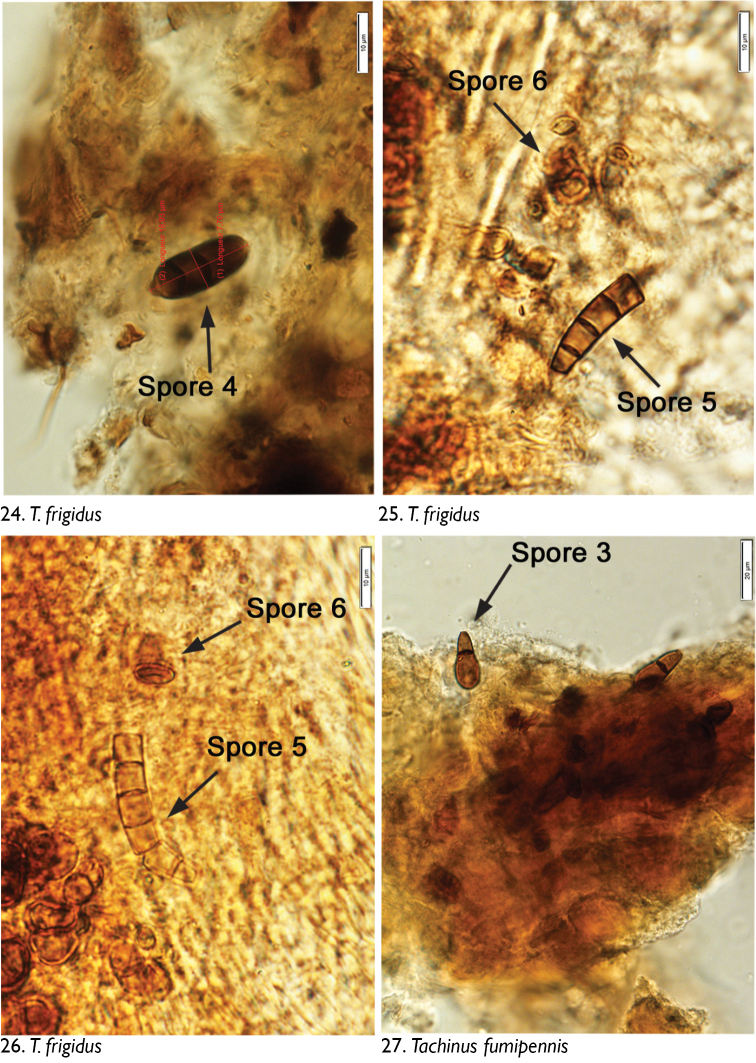
Images of hindgut content of the following rove beetle species: **24–26** *Tachinus frigidus* Erichson **27** *Tachinus fumipennis* (Say).

We observed no cuticle characteristic of arthropods in the guts of any dissected individuals. The only identifiable material was yeasts and fungal spores. Through microscopic observation of spore morphology, we were unable to discriminate among the yeast species, so these were recorded simply as “yeasts” (Figs 12–18, 20, 30, 32, 34, 35). However, at least seven different spore types could be discriminated using microscopic techniques and available taxonomic resources, although they could not be identified with certainty (Figs 17 [spore type 1]; 19 [spore type 2]; 21, 22, 27, 29? [spore type 3]; 23, 24 [spore type 4]; 25, 26, 28 [spore types 5 and/or 6]; and 31, 33 [spore type 7]). Some of these spores are of the following morphology: (long arthrospore fragment, Fig. 17); immature ascomycete cleistothecia or pycnidia, (Fig. 19, spore # 2); and dark walled spores (Figs 31, 33, spore # 7; dark coloured spores, Figs 23, 24, spore # 4). All 10 rove beetle species had yeasts in their hindgut, but spores were found only in the six tachyporine species and were missing in Aleocharinae and Pseudopsinae (Table 2). Yeasts were densely packed in Aleocharinae and Pseudopsinae and less so in remaining species. Spore types 1, 2, 6, and 7 were each found in a single species, while types 3, 4, and 5 were found in *Tachinus fumipennis* and either *Tachinus frigidus* or *Mycetoporus montanus* (Table 2). These two species of *Tachinus* had the most diverse spore diets (three types each).

**Table 2.** Distribution of yeast and spores in different rove beetle species from microscopical observation. Subfamilies are: A, Aleocharinae; P, Pseudopsinae; T, Tachyporinae.

Rove beetle species	Spore Type							
	Yeast	1	2	3	4	5	6	7
*Atheta capsularis* (A)	×							
*Atheta klagesi* (A)	×							
*Oxypoda grandipennis* (A)	×							
*Bryophacis smetanai* (T)	×	×						
*Ischnosoma longicorne* (T)	×		×					
*Mycetoporus montanus* (T)	×			×				
*Tachinus frigidus* (T)	×				×	×	×	
*Tachinus fumipennis* (T)	×			×	×	×		
*Tachinus quebecensis* (T)	×							×
*Pseudopsis subulata* (P)	×							

**Figures 28–31.**
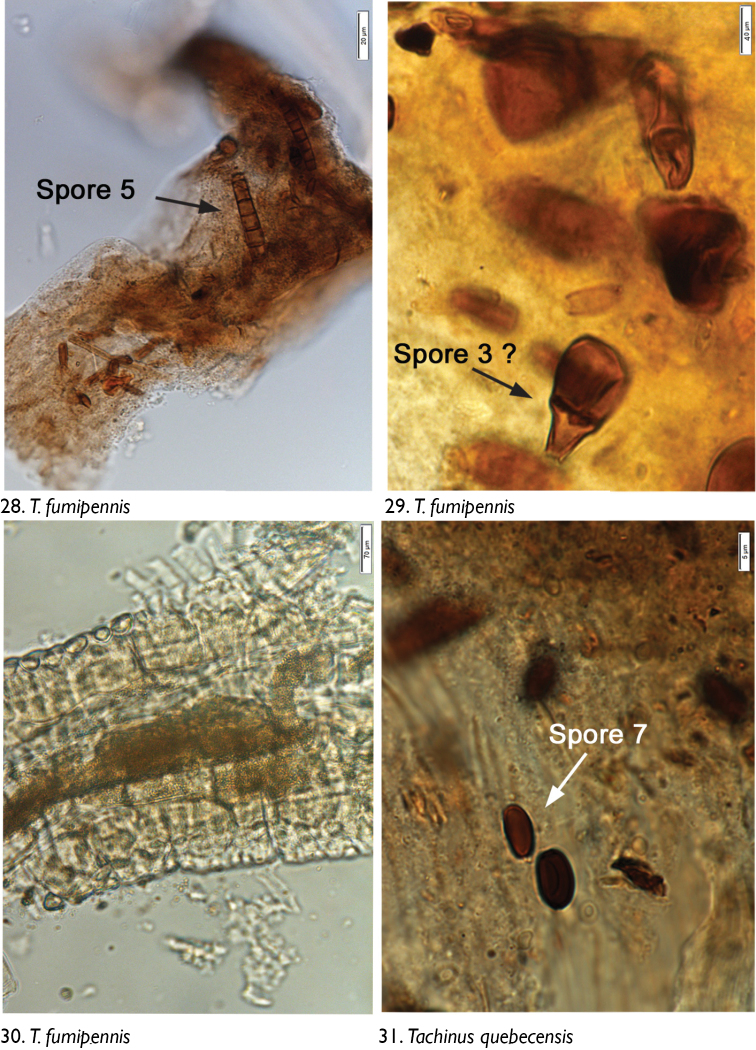
Images of hindgut content of the following rove beetle species: **28–30** *Tachinus fumipennis* (Say) **31** *Tachinus quebecensis* Robert.

**Figures 32–35.**
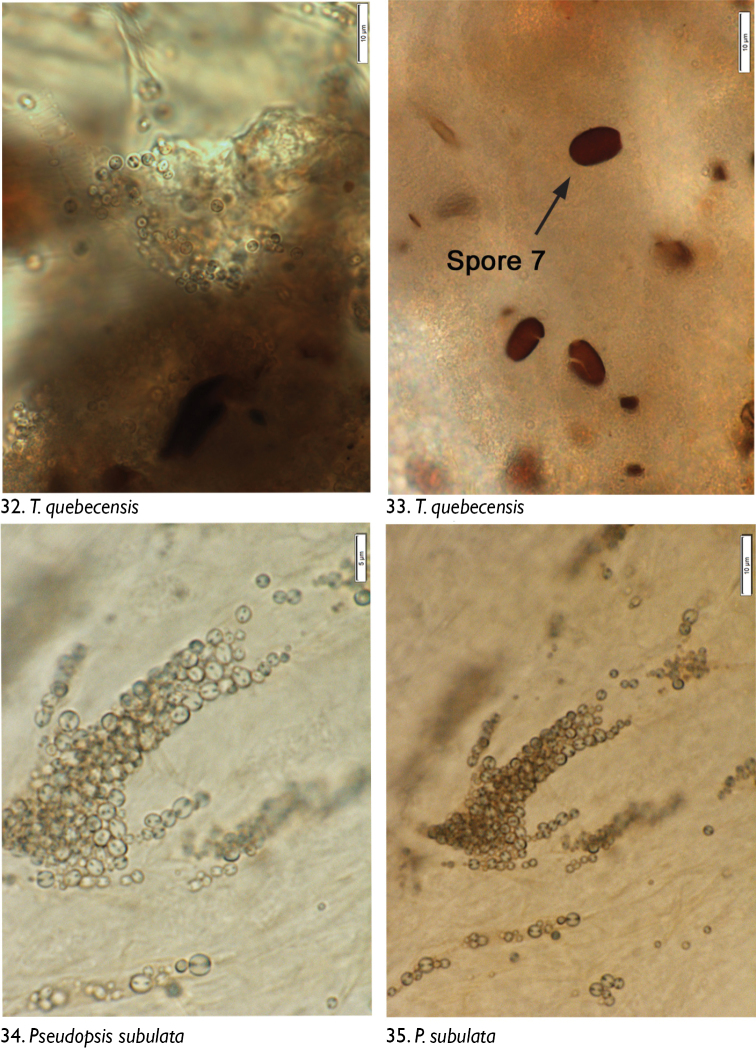
Images of hindgut content of the following rove beetle species: **32–33** *Tachinus quebecensis* Robert **34–35** *Pseudopsis subulata* Herman.

### Molecular analyses

In total, we obtained 186 fungal and bacterial sequences from the 10 species of rove beetles, ranging from 19–33 sequences per species (Table 3). Of these, 134 (72%) could be identified to genus, species, or unnamed clones with high certainty (>90% sequence match) by comparison to sequences in the GenBank and MycoBank databases. Twenty-nine sequences (2 fungal and all 27 bacterial) showed lower levels of sequence similarity (78–90%). We could not match 23 sequences (a range of 0 to 24% unmatched sequences per species, see Table 3).

**Table 3.** Number and identity of genetic sequences extracted from the gut contents of 10 species of Staphylinidae. Accession numbers in brackets follow species name in the first column.

Specific taxon	*Atheta capsularis*	*Atheta klagesi*	*Oxypoda grandipennis*	*Bryophacis smetanai*	*Ischnosoma longicorne*	*Mycetoporus montanus*	*Tachinus frigidus*	*Tachinus fumipennis*	*Tachinus quebecensis*	*Pseudopsis subulata*	Total
**Fungi**											
*Acremonium psammosporum* (GU566287)					2^a^						2^a^
*Alternaria* sp. (GU584946)	1										1
*Aspergillus amstelodami* (HQ728257)	1										1
*Aspergillus versicolor* (AJ937750)										1	1
*Candida cretensis* (HF558653)	1					1	16				18
*Candida mesenterica* (FM178362)	12	8	12	14	5	9	4	20	8		92
*Candida sophiae-reginae* (HQ652073)									1		1
*Candida railenensis* (JX455763)						1					1
*Candida* sp. (AY498864)							1				1
*Cladosporium tassiana* (AF393706)										1	1
*Cryptococcus* (uncultured) (KC753404)		1			2				1		4
*Hypocreales* sp. TR114 (HQ608125)					1						1
*Penicillium spinulosum* (GU566252)					1						1
*Pichia delftensis* (AY923246)								1			1
*Pichia misumaiensis* (U73581)									1		1
*Pichia membranifaciens* (JQ26345)									1		1
*Rhodotorula mucilaginosa* (HQ702343)									1		1
Uncultured fungus clone 50-p12-A5 (HQ267068)		2							5		7
**Insects**											
Ten species from this study		6	8	1	5	10				7	37
**Bacteria**											
*Bradyrhizobium japonicum* (BA000040)				1^b^	2^b^						3^b^
*Serratia marcescens* (CP003942)				4^b^			1^b^	1^b^	2^b^	16^b^	24^b^
**Unmatched sequences**	4	3	1	2	1	1	3	0	0	8	23
**Total**	19	20	21	22	19	22	25	22	20	33	223

^a^ Sequences with 86 to 90% sequence similarity.

^b^ Sequences with 78 to 85% sequence similarity.

In all, we identified 17 fungal taxa in the phyla Ascomycota and Basidiomycota and two bacterial taxa in the phylum Proteobacteria through molecular analysis (Table 3). The number of taxa distinguished from each staphylinid species varied from one in *Oxypoda grandipennis* and *Bryophacis smetanai* to eight in *Tachinus quebecensis*, and averaged 3.3 per species (Table 3). We found yeasts in all of the 10 beetle species studied, with *Candida mesenterica* (Geiger) Diddens & Lodder accounting for 92 sequences and occurring in 9 of the 10 beetle species. The next most commonly identified taxon was the bacterial species *Serratia marcescens* Bizio,which accounted for 24 sequences found in five beetle species. The vast majority of taxa in beetle guts were found in just one (13 taxa) or two (1 taxon) sequences.

## Discussion

Both dissection and molecular analysis of guts strongly suggest that rove beetles in this study may feed primarily on yeasts. Yeasts are ubiquitous (in soil, on decaying plant material including deadwood, and on berries) and they are an important part of the diet of at least some fungivorous beetle species (Suh and Blackwell 2005b). Some species of *Candida* yeasts have close associations with saproxylic insects and are capable of transforming d-xylose and other important components of lignocellulose to ethanol (Wang et al. 2005). However, yeasts within the *Candida mesenterica* clade are associated with many insect groups and are likely indicative of habitat associations rather than being highly specific gut symbionts (Suh and Blackwell 2005a). Yeasts in the *Candida mesenterica* clade, particularly species in its subclade A, are known to be associated with fungal basidiocarps and have previously been isolated from the digestive tracts and body surfaces of six families of basidiocarp-inhabiting beetles, including one unidentified species of Staphylinidae (Suh and Blackwell 2005a).

With the exception of the relatively broad consumption of *Candida mesenterica* yeasts by most species, finer patterns in feeding preferences among rove beetles were difficult to assess. This was partly a result of the limited number of matched sequences for some species, which in turn probably reflects in part the limits of available reference sequences. The limited numbers of sequences obtained in our study could be related to degradation of DNA as the result of suboptimal preservation medium.

The prevalence of other fungi in addition to yeasts and the presence of spores in rove beetle guts was not unexpected, as many rove beetle species are associated with fungi (Campbell 1973, Newton 1984, Newton et al. 2000, Thayer 2005). Spores of at least seven species of fungi were found in the guts of six of the rove beetle species, although it is not known whether the beetles derive nutrition from spores. Many spores have tough walls that enable them to pass through digestive tracts; however, others are certainly digested and some are cracked by the mouthparts to provide nutrition for beetles specializing in spore feeding (Lawrence 1989, Betz et al. 2003). It cannot be determined from available data whether the ingestion of spores by these rove beetles is incidental or intentional. However, the absence of spores in four species, including all three species of Aleocharinae, is notable and raises the question of whether some species do not ingest spores, either because they are unable to, or because they have difficulty finding them in a particular microhabitat.

Although we isolated bacteria less commonly than fungi, we did find them in six beetle species. The second most commonly detected sequence, in fact, was from the bacterium *Serratia marcescens*,which is associated with soils; it may sometimes be pathogenic to insects (Flyg et al. 1980). This species of bacteria is probably not an important food source for rove beetles. It may simply be so common in the soil that incidental ingestion is frequent. The other species of bacterium we isolated, *Bradyrhizobium japonicum*, is a soil-dwelling, nitrogen-fixing species associated with legume plants (Rivas et al. 2009), so seems unlikely to be a food source for rove beetles.

It is notable that no arthropod cuticle or evidence of animal DNA sequences were found in the guts of any of these species despite the fact that predation on arthropods (especially mites, springtails, and smaller insects) is common in the family (Newton et al. 2000, Thayer 2005). Two possible interpretations are that: (1) the adults of these species are entirely fungivorous, or (2) they are predaceous and use preoral digestion, as many staphylinids do (e.g., Evans 1965, Dennison and Hodkinson 1983, Thayer 2005) and specifically as has been hypothesized for *Pseudopsis* (Pseudopsinae) and the entire group of subfamilies to which it belongs (Grebennikov and Newton 2009). The fact that these 10 species represent seven genera and three subfamilies suggests that analysis of gut contents of many more species is needed to provide a better sampling of rove beetle diets. Identification of the many presently unmatched DNA sequences, which could include animal DNA, and ruling out of preoral digestion are also required before carnivory can be excluded with certainty for the species studied here.

In work conducted to characterize rove beetle responses to removal of logging residues following clearcut harvesting in boreal balsam fir forests of Quebec (Work et al. 2013; Klimaszewski, unpublished data), several response patterns were shown by different species. Seven species (*Atheta capsularis*, *Atheta klagesi*, *Bryophacis smetanai*, *Oxypoda grandipennis*, *Tachinus frigidus*, *Tachinus fumipennis* and *Tachinus quebecensis*) were found predominantly or exclusively in uncut forest rather than forest subjected to harvesting treatments. Except for *Tachinus quebecensis*, all of these may feed partly or wholly on basidiocarps, as the predominance of basidiocarp-associated *Candida* spp. in their guts suggests. These species may not persist well in harvested stands because their drier, disturbed conditions are generally far less favourable to mushroom and other sporocarp production (Langor, personal observation). *Tachinus quebecensis* was found only in uncut stands, and had *Candida sophiae-reginae* and *Candida mesenterica* isolated from guts in the present study. However, this beetle species had the highest diversity of ingested fungal species, five of them unique to it, and possibly one or more of these represent important food sources that are absent (or rare) in harvested stands, although it is also possible that it is predaceous and all fungi are incidental.

*Pseudopsis subulata* was the most common species to show a strong affinity for disturbed stands, specifically stands subjected to whole tree harvesting, although it was also found in uncut stands and in stands subjected to harvesting with debris left behind (Work et al. 2013). Interestingly, this is the only rove beetle species that did not have *Candida* spp. in its gut; however, its gut was typically packed with other yeasts that could not be identified. Perhaps these yeasts have a strong association with disturbed and open habitats.

*Mycetoporus montanus* was not collected during the first year of the study (Work et al. 2013), but it was common during the second year, when it was collected almost exclusively in harvested treatments (Klimaszewski, unpublished data). It appears that this species moved into disturbed stands and multiplied rapidly, taking advantage of food or breeding sites that became more available in such stands. Although *Mycetoporus montanus* had *Candida mesenterica* in its gut, it also had a large variety of other organisms that could not be identified, some of which may be the primary food source for this species.

*Ischnosoma longicorne* was commonly found in both uncut and disturbed stands (Work et al. 2013). This species had a high diversity of species in its gut (six fungal and one bacterium species), which may indicate a broad diet and, therefore, a capacity to succeed in many habitat types.

Feeding associations between rove beetles and yeasts provide some insight into potential mechanisms by which biomass harvesting may impact rove beetles. Our results may suggest that dominant rove beetles are feeding on yeasts and other fungi that may or may not be directly associated with sporocarps growing on deadwood substrates. It is important to understand the complexity of factors linking the studied beetles to biomass removal treatments. The removal of additional forest biomass may be affecting beetles not only via potential food linkages, but also by other non-trophic mechanisms such as changes in physical conditions following the removal of the forest overstory (Work et al. 2013).

In addition to characterizing food sources for some abundant species of rove beetles, many of which are good ecological indicators, our work provides some possible explanations for beetle response patterns in the wake of forest disturbance. The relatively easy application of DNA sequencing to gut contents and the steadily increasing wealth of sequence data available to serve as an identification resource means that these techniques can now be readily applied in disturbance ecology research to investigate species response patterns and habitat preferences. We encourage broader use of this approach to support future work.

## Appendix A

**Biological notes about some taxa found in staphylinid guts**

**(Fungal classification below is from Mycoses Study Group (2007))**

**Kingdom FUNGI**

**Phylum ASCOMYCOTA**

Ascomycota, also known as sac fungi, is a sister group of the Basidomycota. This group contains the majority of fungi, including yeasts.

**Saccharomycetales: Saccharomycetaceae: *Candida***

*Candida* is a yeast and the most common cause of opportunistic mycoses worldwide. It is also a frequent colonizer of human skin and mucous membranes. It is also a pathogen and a colonizer, found on leaves, flowers, in water, and in soil. While most *Candida* species are mitosporic, some have a known teleomorphic state and produce sexual spores.

**Saccharomycetales: Saccharomycetaceae: *Cladosporium***

*Cladosporium* is a dematiaceous (pigmented) mould widely distributed in the air and rotten organic material, and frequently isolated as a contaminant in food. Some species are predominant in tropical and subtropical regions. Some *Cladosporium* species are isolated from fish and are associated with infections.

**Saccharomycetales: Endomycetaceae: *Pichia***

*Pichia* is a teleomorph that produces ascospores. The anamorphs of the *Pichia* species are various *Candida* species. The connection of *Candida* species with their corresponding *Pichia* teleomorphs is based on observation of the ascospores produced by the *Candida* isolate or, more specifically, on the 28S gene sequence data. *Pichia ohmeri* was initially isolated from cucumber brine and is commonly used in the food industry for fermentation in pickles, rinds, and fruits. Clinically, *Pichia* is generally considered to be a contaminant. However, some *Pichia* species are now recognized as clinically significant opportunistic pathogens.

**Eurotiales: Trichocomaceae: *Aspergillus***

*Aspergillus* is a filamentous, cosmopolitan and ubiquitous fungus found in nature. It is commonly isolated from soil, plant debris, and indoor air environment. While a teleomorphic state has been described for only some of the *Aspergillus* species, others are accepted to be mitosporic, without any known sexual spore production.

**Pleosporales: Pleosporaceae: *Alternaria***

*Alternaria* is a cosmopolitan dematiaceous (pigmented) fungus commonly isolated from plants, soil, food, and indoor air environment. The production of melanin-like pigmentation is one of its major characteristics. Its teleomorphic genera are *Clathrospora* and *Leptosphaeria*.

**Hypocreales: Hypocreaceae: *Acremonium***

*Acremonium* contains, cosmopolitan filamentous fungi commonly isolated from plant debris and soil. The sexual state of *Acremonium* is not well-defined. Thus, it is classified among the deuteromycetes group of fungi by some authorities. Others prefer to include it in the phylum Ascomycota, since its structural properties are similar to those of this group.

**Phylum BASIDIOMYCOTA**

Basidiomycota contains a wide variety of organisms. It is estimated that there are about 30,000 species in this group. While it is best known for fruiting bodies such as mushrooms, puffballs, and bracket fungi, it also contains microscopical fungi. These include rust and smut fungi, which are both parasites. Basidiomycota fungi are considered to be the most evolutionarily derived of all fungal phyla. Like Ascomycota, the Basidiomycota also contains some forms of yeast. Therefore, the organisms within this classification can be either unicellular or multicellular. There are three major groups within this classification: Urediniomycetes, which includes rusts and other taxa; Ustilaginomycetes, which are largely composed of smuts; and Hymenomycetes, which are composed of mushrooms and jelly fungi.

**Sporidiales: Sporidiobolaceae: *Cryptococcus***

*Cryptococcus* is an encapsulated yeast. Following its first identification in nature from peach juice samples, the major environmental sources of *Cryptococcus neoformans* have been shown to be either soil contaminated with pigeon droppings (*Cryptococcus neoformans* var. *neoformans*) or eucalyptus trees and decaying wood forming hollows in living trees (*Cryptococcus neoformans* var. *gattii*). *Cryptococcus neoformans* var. *gattii* was also isolated from goats with pulmonary disease.

**Sporidiales: Sporidiobolaceae: *Rhodotorula***

*Rhodotorula* is a yeast found in air, soil, lakes and ocean water, and dairy products. It may colonize plants.

**Kingdom EUBACTERIA**

**Phylum: PROTEOBACTERIA**

**Enterobacteriales: Enterobacteriaceae: *Serratia***

*Serratia marcescens* is a motile, short, rod-shaped, gram-negative, facultative anaerobe bacterium classified as an opportunistic pathogen. *Serratia marcescens* was first thought to be harmless (non-pathogenic). Optimally, *Serratia marcescens* grows at 37°C, but it can grow at temperatures that range from 5 to 40°C. It grows at pH levels that range from 5 to 9. *Serratia marcescens* is well known for the red pigmentation it produces, called prodigiosin.

**Rhizobiales: Bradyrhizobiaceae: *Bradyrhizobium***

Members of this genus, including *Bradyrhizobium japonicum*, are gram-negative soil bacteria that fix nitrogen and are commonly associated with legume plants.

## Appendix B

**Table:** Hyperlinks for microorganism identification.

	NCBI Taxonomy Browser	MycoBank
**FUNGI, Ascomycota**		
**Saccharomycetales**		
*Candida cretensis*	http://www.ncbi.nlm.nih.gov/Taxonomy/Browser/wwwtax.cgi?id=268492	http://www.mycobank.org/BioloMICS.aspx?Table=Mycobank&Rec=432161&Fields=All
*Candida mesenterica*	http://www.ncbi.nlm.nih.gov/Taxonomy/Browser/wwwtax.cgi?mode=Info&id=45568&lvl=3&lin=f&keep=1&srchmode=1&unlock	http://www.mycobank.org/BioloMICS.aspx?Table=Mycobank&Rec=106492&Fields=All
*Candida sophiae-reginae*	http://www.ncbi.nlm.nih.gov/Taxonomy/Browser/wwwtax.cgi?mode=Info&id=45593&lvl=3&lin=f&keep=1&srchmode=1&unlock	http://www.mycobank.org/BioloMICS.aspx?Table=Mycobank&Rec=105324&Fields=All
*Davidiella tassiana*	http://www.ncbi.nlm.nih.gov/Taxonomy/Browser/wwwtax.cgi?mode=Info&id=29918&lvl=3&lin=f&keep=1&srchmode=1&unlock	http://www.mycobank.org/BioloMICS.aspx?Table=Mycobank&Rec=411246&Fields=All
*Pichia* sp.	http://www.ncbi.nlm.nih.gov/Taxonomy/Browser/wwwtax.cgi?mode=Info&id=4925&lvl=3&lin=f&keep=1&srchmode=1&unlock	http://www.mycobank.org/BioloMICS.aspx?Table=Mycobank&Rec=97263&Fields=All
*Pichia delftensis*	http://www.ncbi.nlm.nih.gov/Taxonomy/Browser/wwwtax.cgi?mode=Info&id=324739&lvl=3&lin=f&keep=1&srchmode=1&unlock	http://www.mycobank.org/BioloMICS.aspx?Table=Mycobank&Rec=106343&Fields=All
*Pichia misumaiensis*	http://www.ncbi.nlm.nih.gov/Taxonomy/Browser/wwwtax.cgi?id=131113	http://www.mycobank.org/BioloMICS.aspx?Table=Mycobank&Rec=105848&Fields=All
*Pichia membranifaciens*	http://www.ncbi.nlm.nih.gov/Taxonomy/Browser/wwwtax.cgi?mode=Info&id=4926&lvl=3&lin=f&keep=1&srchmode=1&unlock	http://www.mycobank.org/BioloMICS.aspx?Table=Mycobank&Rec=108031&Fields=All
**Eurotiales**		
*Aspergillus amstelodami*	http://www.ncbi.nlm.nih.gov/Taxonomy/Browser/wwwtax.cgi?id=5054	http://www.mycobank.org/BioloMICS.aspx?Table=Mycobank&Rec=9994&Fields=All
*Aspergillus versicolor*	http://www.ncbi.nlm.nih.gov/Taxonomy/Browser/wwwtax.cgi?mode=Info&id=46472&lvl=3&lin=f&keep=1&srchmode=1&unlock	http://www.mycobank.org/BioloMICS.aspx?Table=Mycobank&Rec=2780&Fields=All
*Penicillium spinulosum*	http://www.ncbi.nlm.nih.gov/Taxonomy/Browser/wwwtax.cgi?id=63822	http://www.mycobank.org/BioloMICS.aspx?Table=Mycobank&Rec=19325&Fields=All
**Pleosporales**		
*Alternaria* sp.	http://www.ncbi.nlm.nih.gov/Taxonomy/Browser/wwwtax.cgi?mode=Info&id=1320240&lvl=3&lin=f&keep=1&srchmode=1&unlock	http://www.mycobank.org/BioloMICS.aspx?Table=Mycobank&Rec=31788&Fields=All
**Hypocreales**		
*Hypocreales* sp. TR114	http://www.ncbi.nlm.nih.gov/Taxonomy/Browser/wwwtax.cgi?id=929379	
*Acremonium psammosporum*	http://www.ncbi.nlm.nih.gov/Taxonomy/Browser/wwwtax.cgi?mode=Info&id=745571&lvl=3&lin=f&keep=1&srchmode=1&unlock	http://www.mycobank.org/BioloMICS.aspx?Table=Mycobank&Rec=485&Fields=All
**FUNGI, Basidiomycota**		
*Cryptococcus* (uncultured)	http://www.ncbi.nlm.nih.gov/Taxonomy/Browser/wwwtax.cgi?id=526442	http://www.mycobank.org/BioloMICS.aspx?Table=Mycobank&Rec=54225&Fields=All
*Rhodotorula mucilaginosa*	http://www.ncbi.nlm.nih.gov/Taxonomy/Browser/wwwtax.cgi?id=5537	http://www.mycobank.org/BioloMICS.aspx?Table=Mycobank&Rec=107159&Fields=All
**BACTERIA, Protobacteria**		
**Enterobacteriales**		
*Bradyrhizobium japonicum*	http://www.ncbi.nlm.nih.gov/Taxonomy/Browser/wwwtax.cgi?mode=Info&id=375&lvl=3&lin=f&keep=1&srchmode=1&unlock	
*Serratia marcescens*	http://www.ncbi.nlm.nih.gov/Taxonomy/Browser/wwwtax.cgi?mode=Info&id=615&lvl=3&lin=f&keep=1&srchmode=1&unlock	
